# Supplementary material for: Causal Relationship between Aging and Anorexia Nervosa: A White-Matter-Microstructure-Mediated Mendelian Randomization Analysis
Source: Biomedicines. 2024 Aug 16;12(8):1874. doi: 10.3390/biomedicines12081874 (PMC11351342; doi:10.3390/biomedicines12081874)
Supplement: Supplementary file 1 [file biomedicines-12-01874-s001.zip › Supplementary Figure/Figure S9/Leaveoneout plot of MR analysis for ubm-b-1815 to AN.pdf]

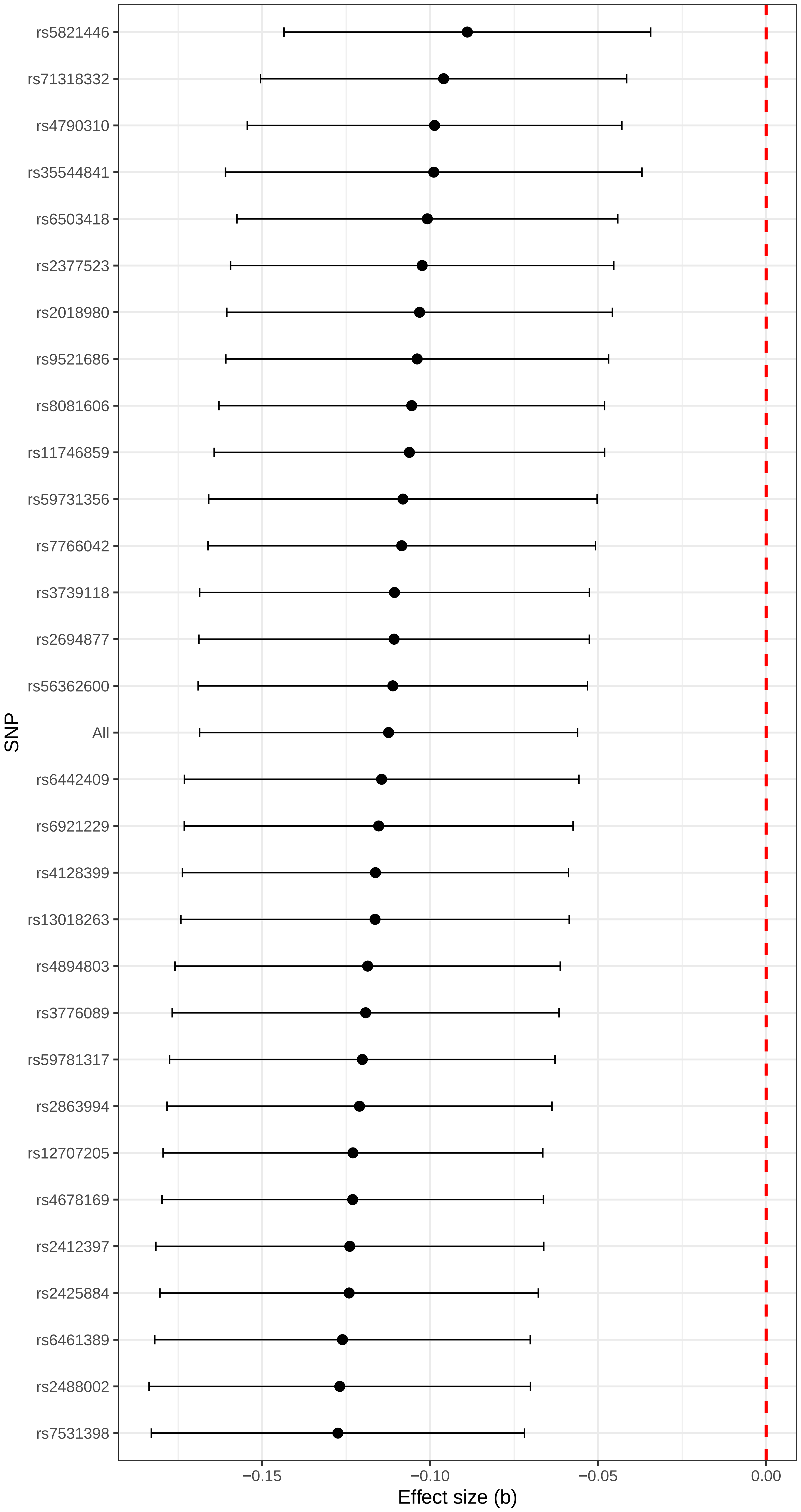

rs5821446
rs71318332
rs4790310
rs35544841
rs6503418
rs2377523
rs2018980
rs9521686
rs8081606
rs11746859
rs59731356
rs7766042
rs3739118
rs2694877
rs56362600
All
rs6442409
rs6921229
rs4128399
rs13018263
rs4894803
rs3776089
rs59781317
rs2863994
rs12707205
rs4678169
rs2412397
rs2425884
rs6461389
rs2488002
rs7531398
SNP
−0.15
−0.10
−0.05
0.00
Effect size (b)
